# Emotional modelling and classification of a large-scale collection of scene images in a cluster environment

**DOI:** 10.1371/journal.pone.0191064

**Published:** 2018-01-10

**Authors:** Jianfang Cao, Yanfei Li, Yun Tian

**Affiliations:** 1 Department of Computer Science & Technology, Xinzhou Teachers University, Xinzhou, China; 2 School of Computer Science & Technology, Taiyuan University of Science and Technology, Taiyuan, China; Maharshi Dayanand University, INDIA

## Abstract

The development of network technology and the popularization of image capturing devices have led to a rapid increase in the number of digital images available, and it is becoming increasingly difficult to identify a desired image from among the massive number of possible images. Images usually contain rich semantic information, and people usually understand images at a high semantic level. Therefore, achieving the ability to use advanced technology to identify the emotional semantics contained in images to enable emotional semantic image classification remains an urgent issue in various industries. To this end, this study proposes an improved OCC emotion model that integrates personality and mood factors for emotional modelling to describe the emotional semantic information contained in an image. The proposed classification system integrates the k-Nearest Neighbour (KNN) algorithm with the Support Vector Machine (SVM) algorithm. The MapReduce parallel programming model was used to adapt the KNN-SVM algorithm for parallel implementation in the Hadoop cluster environment, thereby achieving emotional semantic understanding for the classification of a massive collection of images. For training and testing, 70,000 scene images were randomly selected from the SUN Database. The experimental results indicate that users with different personalities show overall consistency in their emotional understanding of the same image. For a training sample size of 50,000, the classification accuracies for different emotional categories targeted at users with different personalities were approximately 95%, and the training time was only 1/5 of that required for the corresponding algorithm with a single-node architecture. Furthermore, the speedup of the system also showed a linearly increasing tendency. Thus, the experiments achieved a good classification effect and can lay a foundation for classification in terms of additional types of emotional image semantics, thereby demonstrating the practical significance of the proposed model.

## Introduction

With the rapid development of network technology and the popularization of image capturing devices, the quantity of available digital images is exploding. Faced with these colossal quantities of image data, it is becoming increasingly difficult for people to retrieve the particular images they require. This is because when people retrieve images, they do not rely only on the low-level visual features of those images. Images can contain a wealth of emotions, and most of the time, successful image retrieval depends on people’s emotional semantic understanding of the images of interest. Therefore, the classification of images according to emotional semantics is of very important practical significance, and the study of emotional semantic access to images has broad application prospects [1]. In real life, scene images constitute the most common and most widely used type of image data, and the number of such images on the Internet is continuously increasing. Images can be objects for people to appreciate and can regulate people’s moods. Extensive amounts of scene image materials are also used when designing advertisements or websites and when conducting other business activities. Therefore, achieving the emotional modelling and classification of massive collections of scene images to improve users’ browsing and retrieval speeds is an urgent problem.

Since the late 1990s, research on emotional semantic image analysis has undergone rapid development. Researchers have studied the emotional identification of various kinds of image data and have achieved a number of accomplishments. From the psychological perspective, Mao et al. used a two-dimensional mathematical model of fluctuation to develop an image fluctuation analysis method, demonstrating that an image that conforms to the “1/f fluctuation” law is typically considered harmonious and beautiful [2]. Naber et al. studied the relationship between low-level visual features and emotional semantics through the detection and recognition of animals in natural scene images [3]. Kuzinas explored the stability of the relationship between the basic attributes of an image and the emotional associations it evokes [4]. Truelove first studied the association between energy images and people’s cognitive emotions and found that coal and nuclear images tend to evoke negative emotions, natural images produce neutral emotions, and wind images draw out positive emotions [5]. The above studies have found that from the psychological perspective, there is a correlation between images and human emotion. With the development of artificial intelligence, researchers have begun to study how to use computers for emotional modelling. Li et al. constructed a mood and emotion attenuation function based on a study of the relationship between personality and the deterioration of mood, and they obtained data concerning the interactions between various emotions in a fluctuating emotional state as the basis for a multi-layer affective model [6]. Targeting social network images, Wang et al. first verified the existence of the image emotion phenomenon, proposed an emotion model based on a probabilistic factor graph, and then analysed and evaluated the effectiveness of the proposed model using the Flickr network as an example [7]. Later, these researchers applied emotion modelling to the understanding of digital images and explored the extraction of image emotion features, emotional semantic classification, retrieval and other topics. Cho and Lee used the discrete wavelet transform method to extract image features and then realized the retrieval of emotional images by means of an interactive genetic algorithm [8]. Liu et al. proposed a semantic clustering scheme based on local features, which greatly reduced the search space for image retrieval and improved its efficiency [9]. Li et al. proposed an emotion classification method for home design images based on colour features and established a relational model between colour features and emotional semantics based on the perceptual understanding of colour [10]. They used a Radial Basis Function (RBF) neural classifier to perform style classification of home design images. Li et al. proposed an unsupervised feature selection method based on cross-domain convolutional sparse autoencoders and realized the semantic emotion analysis of textile-type images [11]. Zhang et al. proposed a Bayesian multiple-kernel learning algorithm for emotion classification and retrieval and verified the effectiveness of the algorithm on a small sample set [12]. Cao et al. proposed an automatic method for the semantic annotation of scene images based on fuzzy theory and achieved good experimental results using AdaBoost combined with a backpropagation (BP) neural network algorithm to achieve automatic annotation [13]. To address emotional issues related to music and images in cross-media retrieval, Xing et al. presented an optimized model for music/image emotion identification based on the Differential Evolutionary-Support Vector Machine (DE-SVM) algorithm and realized an emotion-driven cross-media retrieval system [14]. To investigate the impact of video sequence images on human audiences, Chang et al. designed a real-time video emotion retrieval scheme based on image sequence features, which can be applied to movies, computer games, video conferences and other video scenarios [15]. Yun et al. used a 3D Gabor database to extract 3D geometric information on facial expressions and colour/density information to construct visual feature vectors, based on which they proposed an improved kernel canonical correlation analysis (IKCCA) algorithm for identifying the emotional state of a human being [16]. Most research on emotional semantics has focused on the establishment of an emotion model and the analysis and understanding of small sets of sample data. However, the key problem in building an emotion model is to establish a vocabulary for emotional expression. At present, researchers tend to define their own emotional vocabularies targeting their own studied images based on their own ideas, and there is no uniform standard, which leads to a lack of generality in their research methodologies. Furthermore, with the increasing volumes of available multimedia data, including images, the efficiency of traditional algorithms for image emotion analysis is waning, and consequently, emotional semantic image analysis is facing new challenges in the era of big data.

The popular MapReduce framework on the Hadoop platform is a parallel computing model oriented towards distributed environments. It provides a complete programming interface for developers, who are not required to understand the structure of the entire computer system. Therefore, it has become a current focus of research involving parallel algorithm design, and it is being applied in an increasing number of fields [17]. Chen et al. proposed a distributed traffic flow forecasting method based on the MapReduce parallel programming model for the rapid handling of the ever-increasing amounts of traffic flow data [18]. The processing of large data sets presents a challenge for traditional clustering algorithms; to address this problem, Kim et al. used the MapReduce model to design a type of density-based clustering algorithm that is suitable for processing big data and has achieved good experimental results [19]. Attribute reduction is an important problem in research related to rough sets. In the face of large data, traditional attribute reduction algorithms are inadequate. Consequently, Jin et al. proposed a hierarchical attribute reduction algorithm for large-scale data processing, which uses MapReduce to perform tasks in parallel, thereby saving considerable time [20]. Such algorithms using the MapReduce model for parallel processing have greatly improved the operational efficiency of systems when handling large-scale data. In the field of digital image processing, Almeer realized the analysis and processing of a massive collection of remote sensing images using the Hadoop Distributed File System (HDFS) [21]. From the perspective of mass image processing technology, Wiley et al. realized the analysis and processing of astronomical images using Hadoop by converting images into serialized binary files [22]. The number of MapReduce-based applications is gradually increasing, and the field of digital image understanding is no exception. However, there is little literature on the application of MapReduce for the emotional semantic classification of large-scale image data sets. Therefore, if the MapReduce parallel programming model could be used for the parallel design of an adapted version of a traditional algorithm for emotional semantic image classification in the Hadoop cluster environment, it would greatly improve the current situation with regard to the emotional semantic understanding of large-scale digital image collections.

In summary, most methods of image classification and retrieval are based on the low-level visual features of images, and little research on large-scale collections of digital images has been conducted with a high-level focus on emotional semantics. In the current study, scene images were analysed from the perspective of emotional modelling. An emotional model was established by combining users’ real understanding of images with personality, mood and other factors, and the MapReduce parallel programming model was used to design an automatic classification model for application in a cluster environment based on a combination of the Support Vector Machine (SVM) and K-Nearest Neighbour (KNN) algorithms, which are important tools for digital image understanding. This work is expected to facilitate research on the high-level semantic retrieval of images, user-personalized retrieval, and other applications.

## Methods

### Emotional model integrating personality and mood factors

#### OCC emotion model

The OCC emotion model was the first structural model of emotions, proposed by Ortony, Clore, and Collins in 1988 for use in the field of artificial intelligence [23]. It provides an emotion classification scheme that defines 22 basic emotions and uses a consistent cognitive structure for the expression of emotions. This scheme is widely used because its constituent definitions are easy to implement on a computer. The OCC model expresses emotions by means of several functions and emotional rules. *D*(*p*, *e*, *t*) is used to indicate the expectation degree of subject *p* desiring to experience an event *e*. If the event is expected to generate beneficial results, then the function value is positive; otherwise, the function value is negative. Consider the “angry” emotion, for example; *I*_*g*_(*p*, *e*, *t*) is used to indicate a combination of the overall intensity variables (e.g., expectation, realization, and approximation), and *P*_*j*_(*p*, *e*, *t*) represents the probability of generating an “angry” state. Then, the rule for generating “anger” is as follows:

ifD(p,e,t)>0

$$then\;P_{j}(p,e,t)=F_{j}(D(p,e,t),I_{g}(p,e,t))$$

where *F*_*j*_() is the function for expressing anger. Although the above rule does not itself produce an experience of anger or an angry feeling, it can be used to trigger another rule. Thus, if the intensity of “anger” is set to *I*_*j*_ for a given threshold *T*_*j*_, the following can be derived:

$$if\;P_{j}(p,e,t)>T_{j}(p,t)$$

$$then\;I_{j}(p,e,t)=P_{j}(p,e,t)-T_{j}(p,t)$$

$$else\;I_{j}(p,e,t)=0$$

This rule activates the emotion of anger. When the strength exceeds the given threshold, the “angry” emotion is generated, and the strength value obtained can be mapped onto one of several more specific “angry” feelings; for example, “mad” corresponds to a moderate value, whereas “hatred” corresponds to a high value. The makeup of the other emotional rules is similar.

However, although the OCC model considers only a generation mechanism based on the cognitive factors driving emotion, emotion is in fact also affected by personality, mood and other non-cognitive factors. Therefore, inadequacies arise when expressing emotion using only the OCC model.

#### Personality factors

Compared with emotion, personality is a relatively constant variable. There is no unified standard for the description of personality. In this study, the FFM model [24] was adopted, which is the model that is most widely used at present in the psychological field to describe personality. This model divides the characterization of personality into 5 categories: openness, conscientiousness, extraversion, agreeableness, and neuroticism. The specific descriptions are as follows:

Openness: imagination, aesthetics, affection, creativity, intelligence, etc.;

Conscientiousness: competence, fairness, orderliness, responsibility, consciousness, restraint, etc.;

Extraversion: enthusiasm, sociality, decisiveness, sense of adventure, optimism, etc.;

Agreeableness: confidence, straightforwardness, altruism, compliance, humility, empathy, etc.;

Neuroticism: anxiety, hospitality, depression, self-awareness, impulsion, fragility, etc.

In this study, a five-element group was defined to describe a person’s personality traits, where −1 ≤ *O*, *C*, *E*, *A*, *N* ≤ 1.

#### Mood factors

To accurately measure people’s moods, scholars have proposed several measurement theories and methods in the context of psychology. In particular, research shows that the PAD model can effectively measure and explain mood. This model is a three-dimensional measurement model proposed by Mehrabian and Russell in 1974. In this model, mood is divided into the dimensions of Pleasure (P), representing the positive and negative characteristics of an individual’s emotional state; arousability (A), representing the individual’s level of neural physiological activation; and dominance (D), representing the individual’s level of control over the situation and others. The possible values for each dimension lie in the range of [-1, +1]. Based on reference [23], we selected the OCC emotion model, which is partially related to images of natural scenery, as the basis for our model; the mapping relationship between this model and the corresponding values for each dimension of the PAD model is as shown in Table 1.

**Table 1:** Mapping relationship between PAD values and the OCC model.

Emotion type	Pleasure (P)	Arousability (A)	Dominance (D)
Sad	-0.40	-0.20	-0.50
Fear	-0.50	-0.30	-0.70
Hate	-0.60	0.60	0.30
Relaxed	0.20	-0.30	0.40
Angry	-0.51	0.59	0.25
Lost	-0.30	0.10	-0.40
Scared	-0.64	0.60	-0.43
Joyous	0.30	0.10	0.20
Proud	0.40	0.30	0.30
Hopeful	0.20	0.20	-0.10

In this study, a three-element group *M*(*P*, *A*, *D*) was defined to measure a person’s mood features, where −1 ≤ *P*, *A*, *D* ≤ 1.

#### Emotional modelling of scene images

According to reference [25], the mapping relationship between personality and mood is defined as follows:

$$\{\begin{array}{l} P=0.21\times E+0.59\times A+0.19\times N\\ A=0.15\times O+0.30\times A-0.57\times N\\ D=0.25\times O+0.17\times C+0.60\times E-0.32\times A \end{array}$$ (1)

where P, A and D on the left-hand sides of the equations represent the three affective dimensions in the PAD model and O, C, E, A, and N on the right-hand sides represent the 5 personality traits in the FFM model.

Because personality spans a five-dimensional space and mood spans a three-dimensional space, according to Eq (1), the mapping matrix between personality and mood can be derived as follows:

$$T\_M=\left[\begin{array}{ccccc} 0 & 0 & 0.21 & 0.59 & 0.19\\ 0.15 & 0 & 0 & 0.30 & -0.57\\ 0.25 & 0.17 & 0.60 & -0.32 & 0 \end{array}\right]$$

such that

M=T_M×T′ (2)

According to the mapping relationship between the OCC emotion model and the PAD values given in Table 1, the mapping matrix between the OCC model and the PAD values can be derived as follows:

$$Y=\left[\begin{array}{ccc} Y_{11} & Y_{12} & Y_{13}\\ Y_{21} & Y_{22} & Y_{23}\\ \cdots & \cdots & \cdots \\ Y_{10,1} & Y_{10,2} & Y_{10,3} \end{array}\right]$$

Therefore, the improved OCC emotion model integrating personality and mood can ultimately be expressed as follows:

T_M_OCC=Y×M (3)

In this way, a 10 × 1 matrix is obtained for quantifying emotion.

### Large-scale scene image classification

#### Image feature selection and emotional semantic mapping

The visual features of images that are commonly considered include colour, texture and shape. In particular, through observation of the human visual system and in combination with the characteristics of natural scene images themselves, we find that colour plays a crucial role in people’s understanding of the emotional semantics associated with images. For a natural scene image, on the one hand, the image itself is typically extremely irregular, so the extraction of texture and shape features is difficult. On the other hand, these two types of features do not play an obvious role in determining the image’s emotional semantics. Therefore, in this study, colour features were extracted as visual features to reflect the emotional semantics of natural scene images [26]. The HSV (Hue, Saturation, and Value) colour space can effectively reflect human perception of colour [27]; therefore, we chose the HSV space as the working space in which to calculate the colour histogram of each image, and we selected the three colours that accounted for the largest proportion of each image as the main colours of that image.

**Fig 1:**
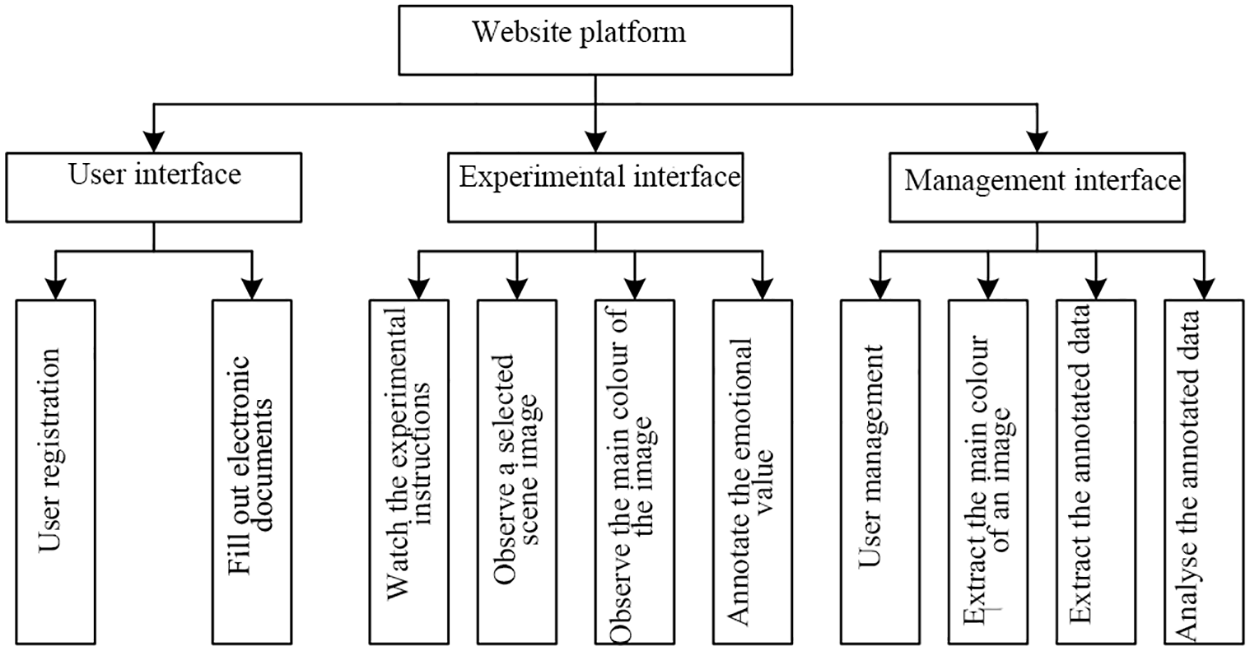
Framework of the behavioural experiment platform.

In daily life and work, when people observe an object, they will tend to have some association with that object. Similarly, when people see a colour image, they will have some association with the colour that applies the most direct stimulation; in other words, they will understand this image in a way that is informed by unique human emotions. For example, when people see an orange colour, they tend to associate it with the golden autumn and rich fruit. Therefore, it is regarded as an abundant, joyous and happy colour, symbolizing warmth, cheerfulness and liveliness. Meanwhile, green is a very beautiful and elegant colour. It is vibrant, symbolizing hope and life. In this study, a summary of the relationships between colours and the improved OCC emotion model was constructed through an experimental investigation. First, we randomly selected 1,000 images from the SUN Database [28] and designed a website as an open-environment experimental platform, whose architecture is shown in Fig 1. The professional psychology software E-Prime was used to present images to subjects, and the specific experimental settings are shown in Table 2. The subjects who participated in the investigation were limited to college students of different years and employees in certain industries, aged between 18 and 45. Before the experiment, the subjects were required to fill out several electronic documents, such as a basic personal information sheet, a personality evaluation scale and an experimental feedback form. During the experiment, the images were randomly presented, and a prompt interface was displayed approximately 1 s before the presentation of each image. After the subject completed the requested choice, there was a rest interface that lasted approximately 1 s to prevent fatigue and deterioration of mood. During the extraction of subject data, the rationality and validity of the data were preliminarily evaluated, and obviously invalid data were removed before statistical analysis. The vocabulary with the highest frequency and the corresponding relationships were summarized, and the statistical survey results are presented in Table 3.

**Table 2:** Experimental scheme for emotional semantic acquisition.

Number of experimental images: 1000				
Emotion model	Emotion type	Annotated value	Manner of annotation	Image display time
OCC emotion model	Sad	0	Click the left mouse button; Annotate one image with one emotion type.	6 s
	Fear	1		
	Hate	2		
	Relaxed	3		
	Angry	4		
	Lost	5		
	Scared	6		
	Joyous	7		
	Pride	8		
	Hopeful	9		

**Table 3:** Mapping between colour and emotion.

Colour	Colour semantic description	OCC emotion word(s)
red	warm, festive, passionate, romantic	joyous, proud
orange	warm, friendly, soft	joyous
yellow	bright, gentle, vivacious	joyous, relaxed
green	hopeful, alive, vital, fresh	hopeful
cyan	vigorous, beautiful	hopeful, relaxed
blue	neat, calm, cold, aloof, sad	sad
purple	romantic, elegant, mysterious, noble	proud
white	monotonous, indifferent, poor	lost
gray	random, old, indifferent	lost, scared
black	serious, death, heavy, horrible	angry, fear, hate

#### SVM-KNN image classification algorithm based on MapReduce

The SVM algorithm is a machine learning method proposed by Vapnik in 1995, based on statistical learning theory and structural risk minimization theory [29]. It is designed for two-category classification, has a good generalization ability, and is suitable for solving nonlinear problems involving high-dimensional data; consequently, it has been widely used in classification, identification, retrieval, and similar tasks. However, Vapnik demonstrated through analysis that in the classification of data into two categories, the SVM algorithm tends to produce some misclassifications in the boundary or overlap area between the two categories. The KNN algorithm is a distance- and example-based non-parametric method, proposed by Cover and Hart in 1968 [30]. Although this algorithm is simple and effective, for each datum to be classified, it is necessary to first calculate the similarity between it and each sample in the space to derive the K nearest adjacent points before the comparison is performed. Therefore, the computational cost of the KNN algorithm is relatively large.

However, we can view an SVM classifier as a nearest neighbour classifier that has only one representative point for each class and combine the SVM and KNN algorithms to generate a new algorithm, namely, the SVM-KNN algorithm [31]. This algorithm selects an appropriate penalty parameter and kernel function for the SVM algorithm and incorporates weight coefficients for the KNN algorithm, which can be derived based on the ratio of the number of samples in a class to the total number of samples. When calculating the distance between a datum to be classified and a sample representing a class, the distance is multiplied by the weight coefficient for this class, and the results are used as the basis for comparison. A class with a larger sample size is assigned a smaller weight, and a class with a smaller sample size is assigned a larger weight, thereby avoiding the undesirable impact on classification caused by an uneven sample distribution. At the same time, the Euclidean distance similarity used in the KNN algorithm is replaced with the cosine similarity of a vector space, which has a more stable effect on classification. This improved SVM-KNN algorithm combines the merits of the two original algorithms and optimizes their performance. Furthermore, it does not require as many calculations as the original KNN algorithm does and simultaneously achieves an improved classification accuracy. The main steps of the algorithm are as follows:

1. A training set of scene image samples is used to train the SVM algorithm; thus, the coefficient ω* and the constant b* of the decision function *f*(*x*) = sgn(ω*x + b*) are derived to obtain the support vector set *D*_*sv*_ and the given threshold *ξ*. In addition, the test data set *D* is preprocessed.
2. If *D* is empty, then execution is stopped; if *D* is not empty, then the datum *x* to be classified is extracted from *D*.
3. The datum *x* to be classified is used to calculate $g(x)=\sum_{i=1}^{n}a_{i}^{*}y_{i}(x_{i}\cdot x)+b^{*}$ to derive *dis* = |*g*(*x*)|.
4. If *dis* > *ξ*, then a suitable kernel function is selected for classification by the SVM algorithm. Finally, *f*(*x*) is exported.
5. If *dis* < *ξ*, then the KNN algorithm is used for classification. The value of *K* is selected, and the datum *x* to be classified is imported. The support vector set *D*_*sv*_ is used as the sample points. In the distance calculations, the weight coefficients are used to balance the samples, and then, the final results are exported.
6. The datum *x*, which has now been classified, is removed from the test data set *D*, and then algorithm returns to Step (2) for continued execution.

With the advent of the era of big data, the amounts of available image data of various types have increased to orders of magnitude of GB, TB, or even PB. The time efficiency and classification accuracy of a classification algorithm with a single-node architecture as described above will decline sharply when applied to such massive amounts of data. Therefore, image classification algorithms with single-node architectures are facing new challenges [32]. To address this issue, the MapReduce parallel programming model was used in this study to adapt the SVM-KNN algorithm to a parallel design.

The basic idea is as follows: First, a large-scale scene image training data set is subjected to segmentation such that its balance is maintained; thus, each segment can be considered to be generally independent, and feature extraction and classification can be performed for the different segments in parallel to reduce the training time of the SVM algorithm. For a small data set that has already been classified, the SMO algorithm [33] is adopted for training to increase the efficiency of deriving the support vector set for each small data set. Each segment is trained to filter out non-support vector points while retaining support vector points. Then, the training results for two segments are combined to form the next input, and this process is iterated until a single support vector set is obtained. Then, this set is judged to determine whether it has reached the desired iterative precision. Once the SVM classifier has been trained, the test data are also subjected to segmentation processing. The data are successively extracted for calculation on their own nodes to derive *dis* for comparison with the given threshold *ξ*, during which each node can independently choose whether to use the SVM algorithm or the KNN algorithm for classification. After the classification of all test data has been completed, the classification results for each node are subjected to unified processing and analysis. Thus, the realization of the parallel SVM-KNN (PSVM-KNN) algorithm requires 4 pairs of Map and Reduce functions, as follows: IterationMapper() and IterationReducer() for the generation of the support vector set, the coefficient w* and the constant b* through iterative training; DisMapper() and DisReducer() for the calculation of *dis*; SVMMapper() and SVMReducer() for the parallel SVM classification functions; and KNNMapper() and KNNReducer() for the parallel KNN classification functions.

Function Iteration ()

{

IterationMapper()

{

split(); //the scene image training set is segmented and sent to each node for processing

while(support vector set>1)

  the support vector set of each segment is calculated;

}

IterationReducer()

{

//the support vector sets output from IterationMapper() in key/value form are paired and processed

if(the final support vector set has reached the desired iterative precision)

return *D*_*sv*_, w*, b*;

  else

 call Iteration();

}

}

Function Dis()

{

DisMapper()

{

  split_dis(); //the test data set is segmented and sent to each node for processing

   for the test data set *D*′ in each segment do

    calculate *dis* in each segment;

}

 DisReducer()

 {

//the support vector sets output by DisMapper() in key/value form are paired and processed

 return *dis*;

  if(*dis* > *ξ*)

   call SVM();

  else

   call KNN();

}

}

Function SVM()

{

 SVMMapper()

 {

  split_SVM(); //the support vector set with *dis* > *ξ* is segmented and sent to each node for processing

  for each *dis* in each segment do

   use the SVM algorithm for classification;

 }

 SVMReducer()

 {

  the results output from SVMMapper() in key/value form are paired and processed;

 }

}

Function KNN()

{

 KNNMapper()

 {

  split_KNN(); //the support vector set with *dis* ≤ *ξ* is segmented and sent to each node for processing

  for each *dis* in each segment do

  {

   add the weight coefficients to recalculate *dis*;

   apply the KNN algorithm calculation;

  }

 }

 KNNReducer()

 {

  the results output from KNNMapper() in key/value form are paired and processed;

 }

}

For the parallel SVM-KNN classification algorithm, the steps of the semantic extraction and classification process for a large-scale collection of scene images are as follows:

1. In accordance with the FFM model, extract human personality traits and calculate the quantized values of the mood factors according to Eq (2).
2. Extract each image’s colour features and obtain the corresponding OCC emotional words according to Table 2. Assign weights of 0.8, 0.6 and 0.35 to the emotional words corresponding to the first, second and third most predominant colours, respectively, according to the proportion of each colour in the colour histogram, based on the results of repeated experiments.
3. Multiply the P, A, and D values of the corresponding emotional words in Table 1 by the derived weights to update the mapping matrix Y.
4. Obtain the quantized emotional values for each image through calculation according to Eq (3) and select the largest value to classify the emotional semantics of the image.
5. Obtain training and test data as described above and use the MapReduce parallel programming model to construct a parallel implementation of the SVM-KNN classification model on the Hadoop platform to realize the automatic emotional semantic classification of scene images.

## Results and discussion

### Experimental environment and data sources

#### Experimental environment

A Hadoop cluster consisting of five computers was used, with one as the Master node and the remaining four as Slave nodes. Each node had a basic configuration consisting of a 4G quad-core processor, 1 TB of hard disk space, and the Ubuntu operating system.

#### Data sources

The experimental data used in this article consisted of images obtained from the SUN Database scene image library (publicly accessible at http://groups.csail.mit.edu/vision/SUN/) combined with images we acquired ourselves. The Sun Database is free to use for researchers in related fields. The library includes 131,067 image samples in 908 scene categories. In total, 20,000 scene images were randomly selected as test samples, and 50,000 scene images were used as training samples. For experimental convenience, all images used in this study were processed to 200 pixels × 200 pixels in size.

### Classification experiment and analysis

#### Classification accuracy

To verify the effectiveness of the algorithm, an experimental comparison was first performed in terms of classification accuracy.

**Fig 2:**
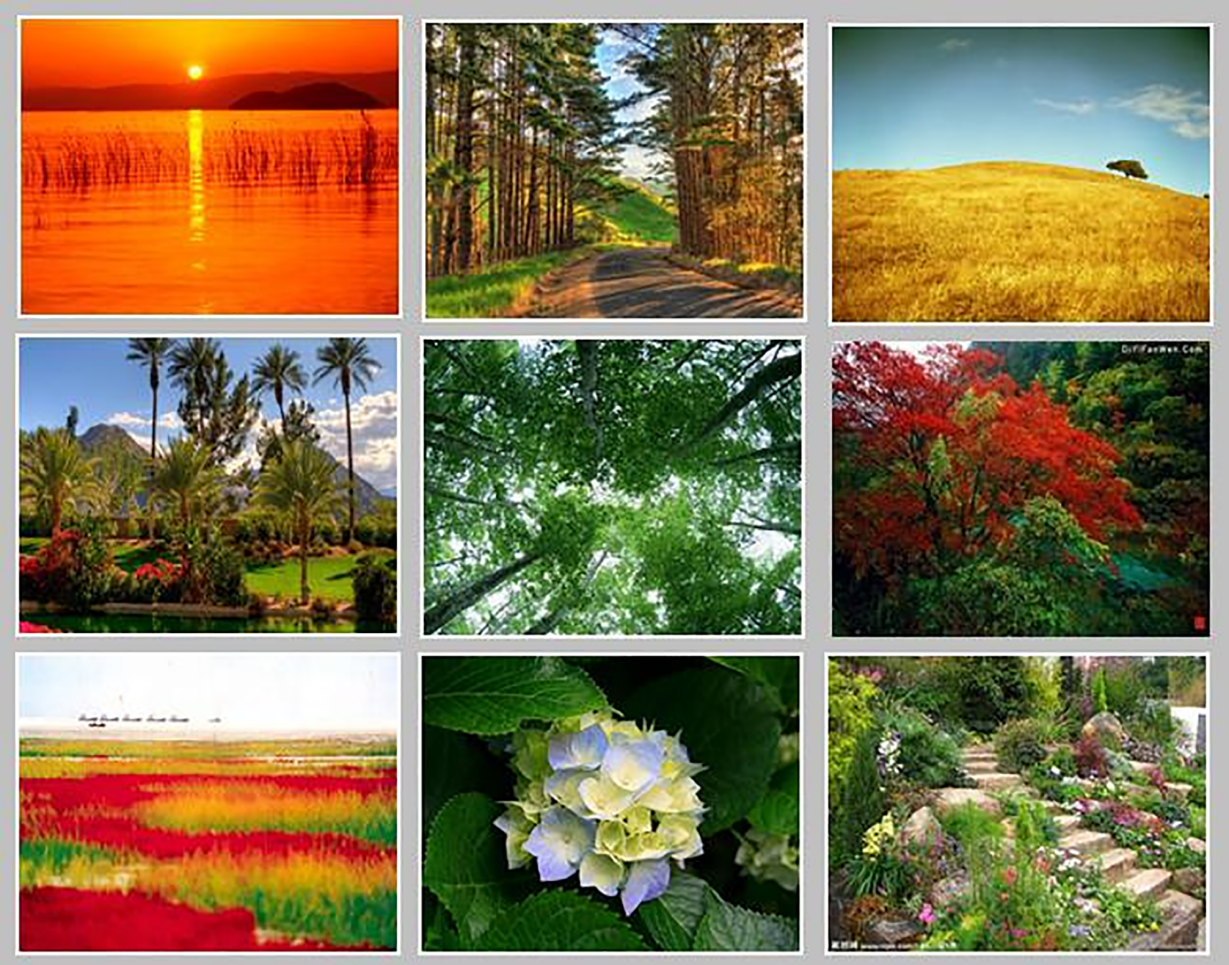
Classification results for extraverted users for the “joyous” and “hopeful” emotions.

Fig 2 shows the classification results for several scene images identified as “joyous” and “hopeful” by users with extraverted personalities. Table 4 lists the classification test accuracies for users of the 5 different personality types for the 20,000-scene-image test sample set in the 10 emotional categories, as obtained using the PSVM-KNN algorithm proposed in this study with a training sample size of 30,000 images.

**Table 4:** PSVM-KNN classification test accuracy.

Emotion type	Openness	Conscientiousness	Extraversion	Agreeableness	Neuroticism
sad	94.6%	92.2%	95.3%	93.1%	91.4%
fear	92.8%	93.2%	93.1%	90.9%	87.6%
hate	85.3%	86.9%	88.5%	79.1%	78.1%
relaxed	98.4%	97.2%	99.2%	95.8%	93.5%
angry	95.1%	94.6%	92.3%	92.2%	89.9%
lost	81.3%	83.5%	78.3%	77.1%	76.6%
afraid	96.4%	96.3%	98.7%	95.4%	90.7%
joyous	99.7%	98.4%	99.3%	96.8%	94.1%
proud	95.4%	97.7%	95.1%	91.3%	83.3%
hopeful	97.7%	95.4%	98.5%	95.4%	90.7%

Fig 2 and Table 4 show that the classification performance of the system is satisfactory; the overall classification accuracy is high. Users with different personalities show overall consistency in their understanding of the same image, but the classification accuracy for users with neurotic personalities is lower, indicating that for the same image, the emotional understanding of users with this personality type is more complex and fluctuates significantly. The system’s classification accuracy is also low for the emotions of “lost” and “hate”, indicating that the emotional understanding of visual elements associated with these emotions is rather fuzzy.

**Fig 3:**
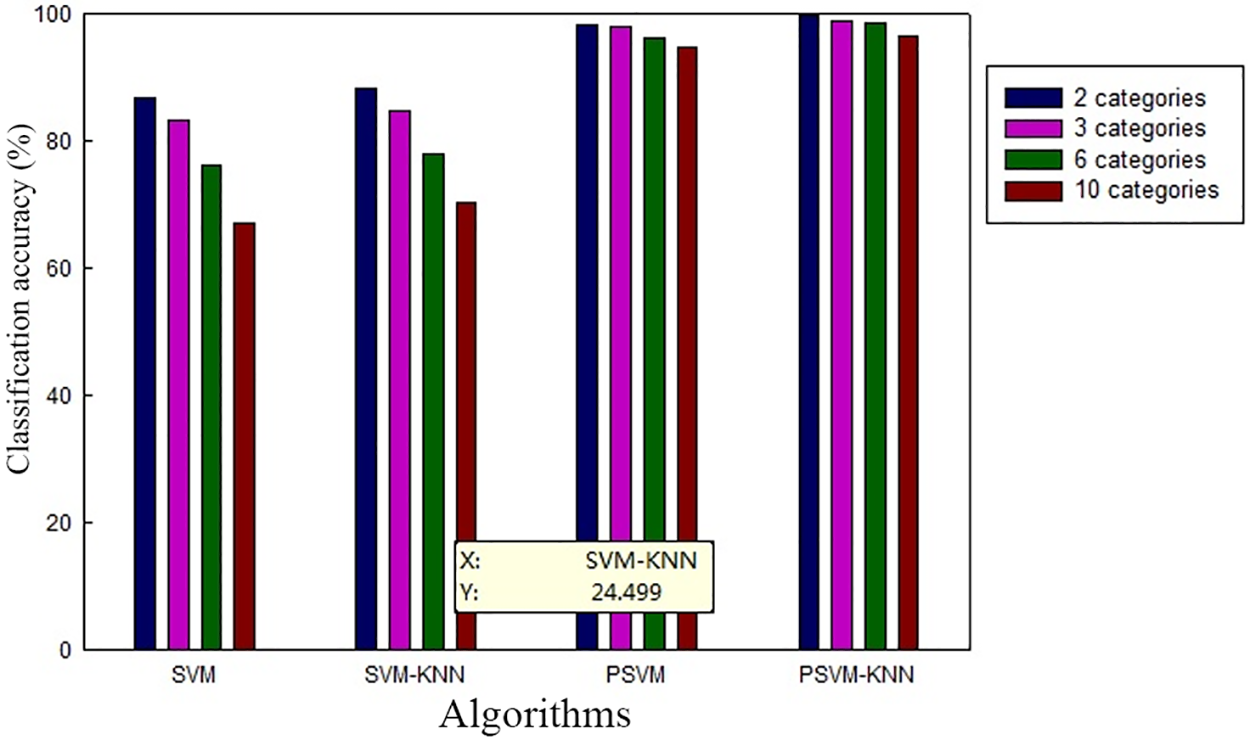
Comparison of the average classification accuracies of different algorithms for different numbers of classification categories.

To further illustrate the classification performance of the PSVM-KNN algorithm proposed in this study, we compared the experimental results of the PSVM-KNN, SVM, and SVM-KNN algorithms and the parallel support vector machine (PSVM) algorithm proposed in reference [34] for different training sample sizes. Table 5 compares the average test accuracies achieved for users with all different personality types using the different algorithms with different training sample sizes for positive emotions such as “joyous”, “proud” and “hopeful”. Fig 3 compares the average classification accuracies of the different algorithms for different numbers of classification categories.

**Table 5:** Comparison of the average classification accuracies of different algorithms for different numbers of training samples.

Number of training samples	SVM	SVM-KNN	PSVM [23]	PSVM-KNN
1000	71.85%	74.39%	82.64%	86.15%
2000	72.94%	76.13%	87.91%	90.45%
5000	77.58%	79.81%	92.49%	95.71%
10000	82.29%	83.96%	95.01%	96.24%
20000	89.37%	91.47%	95.81%	96.83%
50000	91.56%	93.91%	96.15%	97.92%

The results presented in Table 5 show that the classification accuracy increases with an increase in the number of training samples. However, for the SVM and SVM-KNN algorithms, which have a traditional single-node architecture, even though the accuracy is improved, the training time is also very long when the number of training samples is more than 5,000. For the PSVM and PSVM-KNN algorithms, the classification accuracies are higher than those of the SVM and SVM-KNN algorithms, and when the number of training samples reaches 10,000, the classification accuracies of these two parallel algorithms are both above 95%. Of the two, the accuracy of the PSVM-KNN algorithm proposed in this study is higher. Fig 3 shows that an increase in the number of emotion categories considered will cause the classification accuracy to decrease. However, it is obvious that the decreases for the traditional SVM algorithm and the SVM-KNN algorithm based on a single-node architecture are greater, whereas the decreases for the parallel algorithms implemented in a cluster environment are smaller; in particular, the decreases for the PSVM-KNN algorithm proposed in this study are the smallest in magnitude, thereby fully demonstrating the effectiveness of the proposed algorithm.

**Fig 4:**
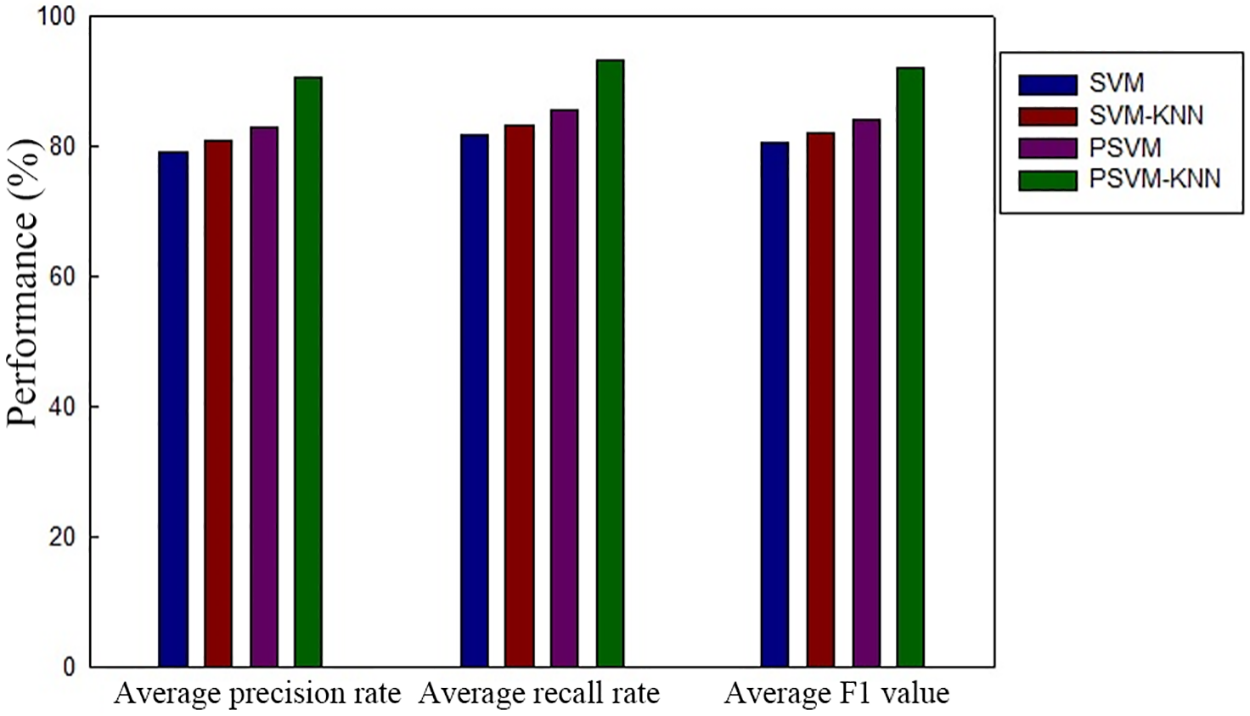
Comparison of the average precision rates, recall rates and F1 values for the different classification algorithms.

**Fig 5:**
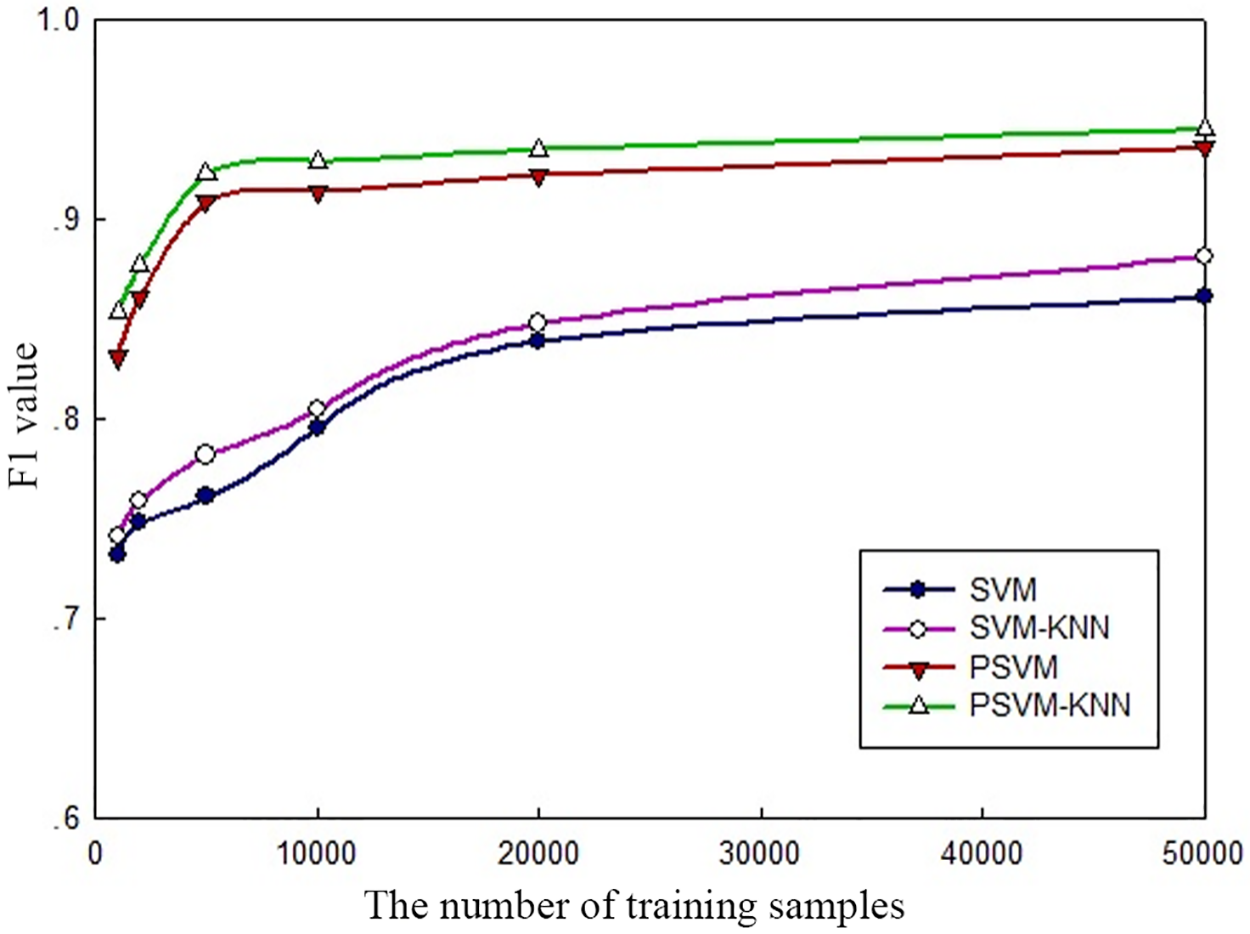
Relationships between the number of training samples and the F1 value for the different classification algorithms.

The precision rate and recall rate are common criteria used to evaluate image classification performance. The precision rate is the proportion of relevant images that the system retrieves relative to all images it finds during one round of analysis and retrieval. The recall rate is the proportion of relevant images the system retrieves relative to all relevant images in the image library during one round of analysis and retrieval. The accuracy rate and recall rate can be combined to calculate F1 = 2 × precision rate × recall rate/(precision rate + recall rate). The F1 value is used as an overall indicator of classification quality. A higher F1 value indicates a better classification effect and a higher classification accuracy. Fig 4 compares the average precision rates, recall rates and F1 values for users with all different personality types for all 10 emotional categories. Fig 5 shows the relationships between the number of training samples and the F1 value for the different classification algorithms.

The results presented in Fig 4 show that because of the use of the MapReduce distributed parallel programming model in a cluster environment, the PSVM and PSVM-KNN algorithms have higher precision rates, recall rates and F1 values than the SVM and SVM-KNN algorithms, which are based on a single-node architecture. Moreover, because the algorithm proposed in this study uses the KNN algorithm to optimize the SVM algorithm’s performance, it achieves a greater advantage in classification performance. Fig 5 shows that for all of the different classification algorithms, the accuracy of their classification results increases with an increase in the number of training samples. However, their rates of increase eventually decline because once the training sample set reaches a sufficient size for a good classification model to be obtained, a further increase in the number of training samples will have no significant impact on the classification performance. Furthermore, the F1 values of the parallel algorithms are significantly higher than those of the algorithms based on the traditional single-node architecture, clearly demonstrating that the parallel approach is suitable for large-scale data processing. As the amount of data to be processed increases, the performance does not exhibit a linear decline. Instead, it tends to reach an optimum.

To further assess the correctness of the method proposed in this paper, we performed an evaluation using Youden′s index. Youden′s index (also called Youden′s J statistic) is a measure for evaluating the authenticity of screening experiments; it is commonly used in the medical field and captures the total performance of a screening method in terms of the missed diagnosis rate and the misdiagnosis rate [35]. For image classification problems, it is a measure of the balance between the precision rate and the recall rate. A larger value of Youden′s index indicates a better screening effect and higher authenticity. The calculation formula is as follows: Youden′s index = recall rate + precision rate– 1 [36].

**Fig 6:**
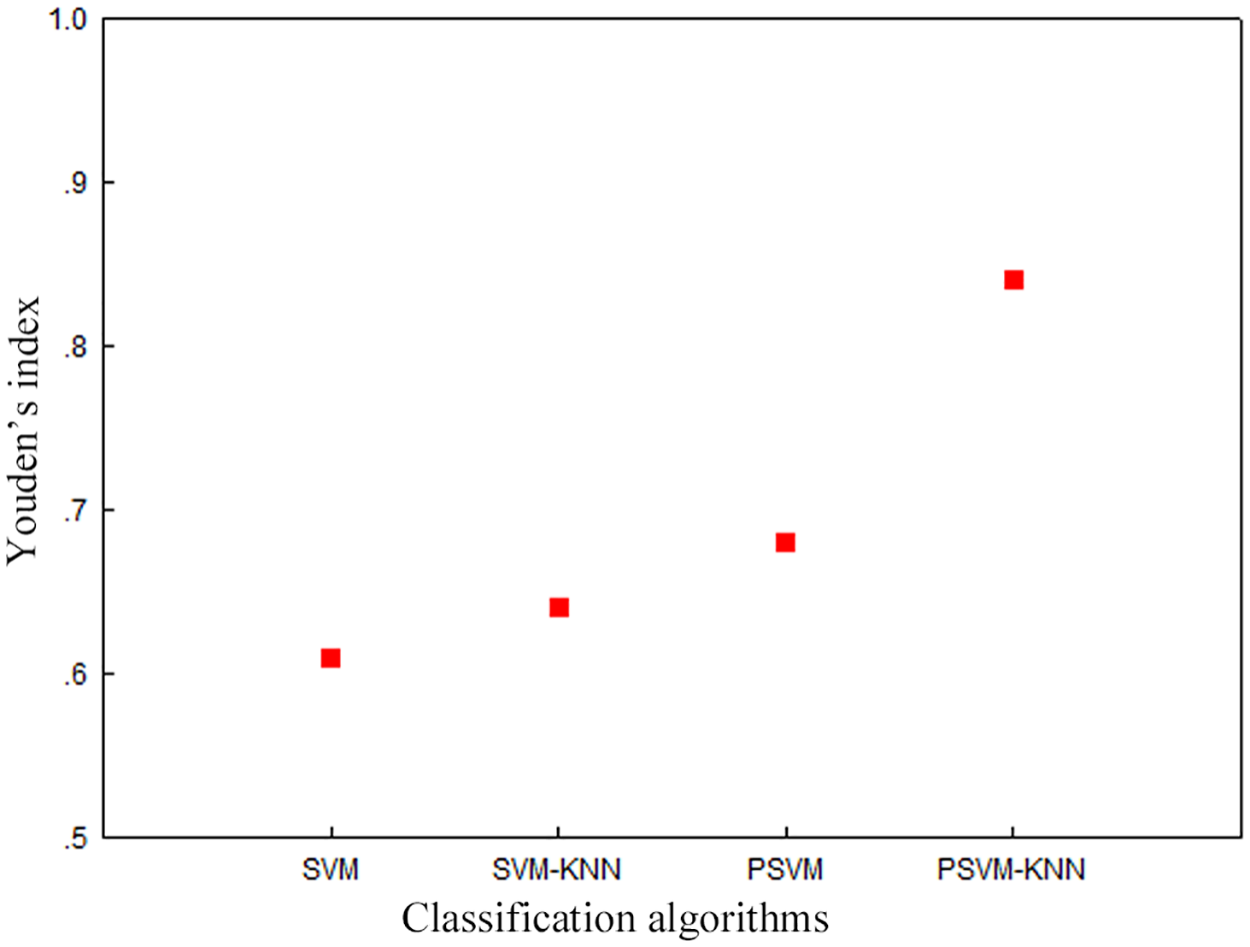
Comparison of the Youden′s index values achieved using different classification algorithms.

Fig 6 compares the experimental results in terms of Youden′s index for the classification results presented in Fig 4.

The results in Fig 6 show that the algorithm proposed in this paper achieves a Youden’s index value of approximately 0.85, which is much higher than the values achieved by the other classification algorithms. This finding further demonstrates that the classification results reported in this paper achieve a good balance between the precision rate and the recall rate and moreover, that the screening effect and authenticity of classification are excellent.

#### Training time

**Fig 7:**
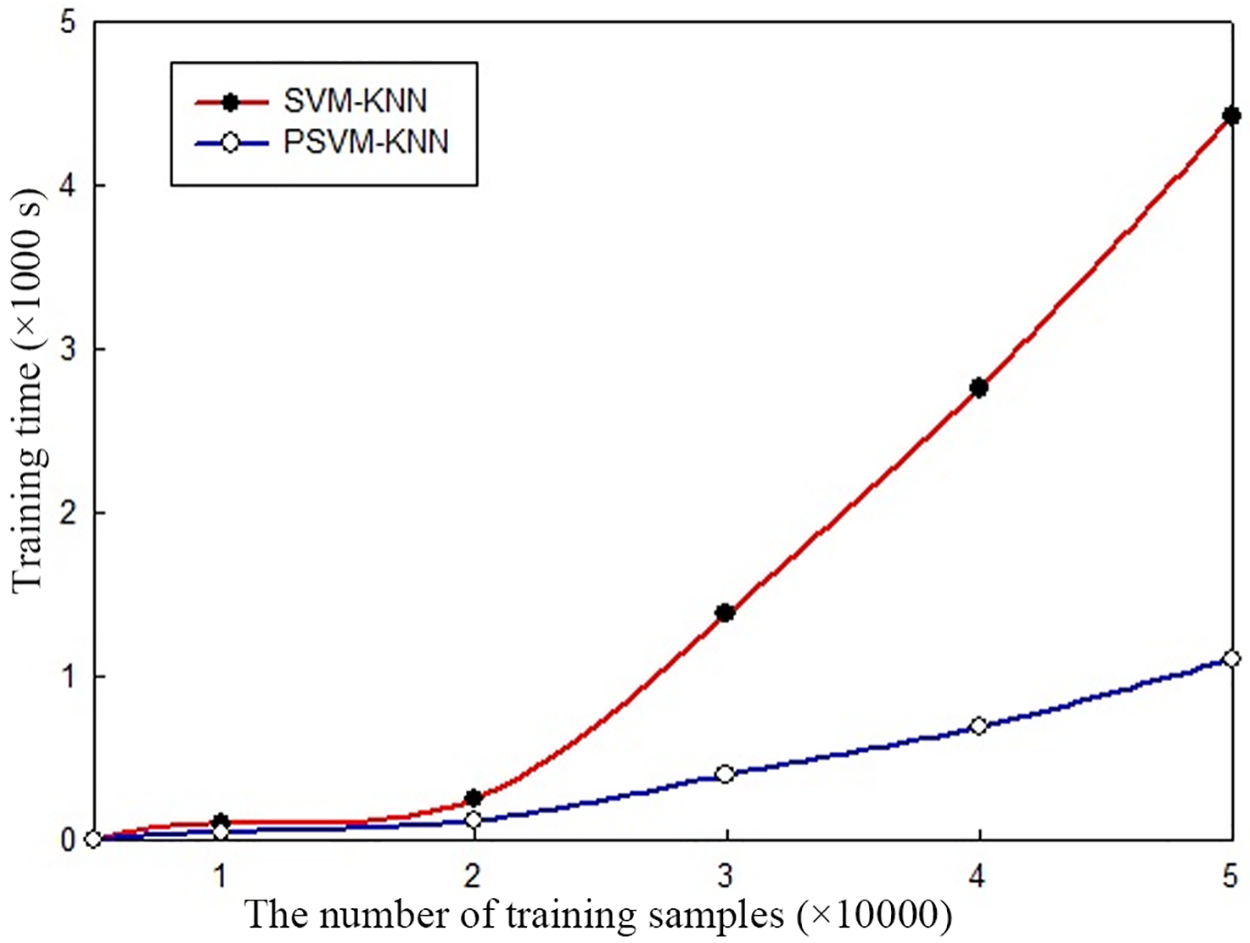
Comparison of training times.

To investigate the time performance of the parallel algorithms in a cluster environment, training time experiments were conducted for the single-node algorithms and the parallel algorithms based on different training sample sizes. However, the training times for the single-node algorithms (SVM and SVM-KNN) were essentially the same, as were the training times for the parallel algorithms (PSVM and PSVM-KNN). Therefore, only the training times for the SVM-KNN and PSVM-KNN algorithms are compared in Fig 7.

As shown in the figure, when the number of training samples is small, the training time for the PSVM-KNN algorithm is nearly identical to that for the SVM-KNN algorithm. However, as the number of training samples increases, the training time for the SVM-KNN algorithm increases exponentially. For larger training sample sizes, given the same number of training samples, the training time for the SVM-KNN algorithm is much longer than that for the PSVM-KNN algorithm on the Hadoop platform. As the number of training samples increases, the time difference becomes more obvious. These findings also demonstrate the power of distributed parallel computing in processing large amounts of data in a cluster environment.

#### System speedup

**Fig 8:**
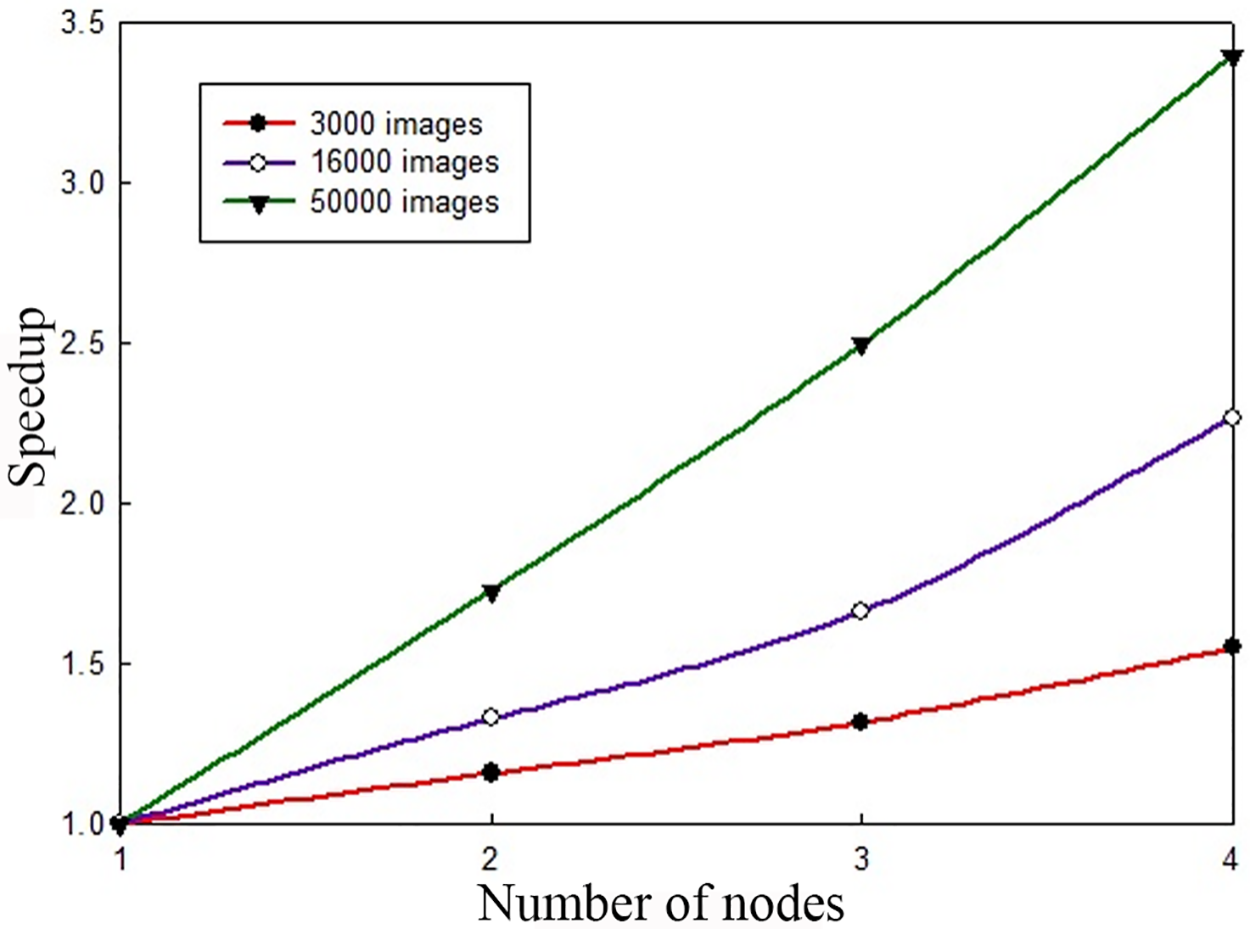
Speedup comparison.

The speedup is the ratio of the running time of a task in a single-node environment to that of the same task in a multi-node environment [37]. It is an important indicator of the efficiency of a parallel algorithm on the Hadoop platform. To verify the performance of the proposed algorithm on the Hadoop platform, the speedup of the algorithm was tested on three data sets containing 3,000, 16,000 and 50,000 images in different emotion categories. The results are shown in Fig 8.

Ideally, the speedup of the system should grow linearly with the number of computer nodes. However, due to communication overhead and load balancing, the speedup does not increase linearly in practice. As seen in Fig 8, when the number of images is small, the speedup of the system increases as the number of nodes increases. However, the rate of increase is not high. As the number of images increases, the growth rate also increases. When the number of images reaches 50,000, the speedup of the system grows almost linearly, thereby providing another demonstration of the superior performance that can be achieved by exploiting the Hadoop cluster environment when handling large-scale data sets.

## Conclusions

The questions of how to give computers the ability to identify and express emotions like human beings and achieve harmonious human-computer interaction are critical challenges facing artificial intelligence today. In this study, the OCC emotion model was integrated with personality and mood factors to explore the relationship between scene images and humans’ subjective understanding of image semantics. Furthermore, a classification model was established based on the SVM-KNN algorithm for implementation in a cluster environment to realize the semantic classification of large-scale collections of scene images. Experimental results show that the proposed PSVM-KNN algorithm has several advantages in addressing the problem of achieving emotional understanding for large-scale collections of scene images. With 50,000 training samples, the classification accuracies for users with various personality types and images in different emotion categories were approximately 95%, yet the training time required was only 1/5 of that required for the corresponding single-node algorithm. Moreover, the system speedup was found to show a linear growth trend, indicating good results.

Human emotions are complex, and the ultimate ideal goal of emotional modelling is to endow computers with emotions. With the arrival of the big data age, the emotional semantic analysis and processing of large amounts of image data has become a hot topic of research. The limitations of this study and possible directions for future research are summarized as follows:

1. Images contain particularly rich semantic contents; consequently, further in-depth and detailed study will be required to determine how to more rationally categorize the emotions associated with images.
2. The number of nodes in the Hadoop cluster should be expanded, and the relevant system parameters should be adjusted to further improve the efficiency of the distributed parallel system.
3. The design of the Map and Reduce tasks in the MapReduce parallel programming model should be optimized, and the communication overhead between the nodes in the cluster environment should be reduced to achieve faster and more accurate evaluation and prediction.

## References

[1] Cao LJ, Wang FL. Robust latent semantic exploration for image retrieval in social media. Neurocomputing 2015; 169: 180–184.

[2] Mao X, Ding YK, Muta I. Analysis of affective characteristics and evaluation on harmonious feeling of image. Acta Electronica Sinica 2001; 29: 1923–1927.

[3] Naber M, Hilger M, Einhäuser W. Animal detection and identification in natural scenes: image statistics and emotional valence. Journal of Vision 2012; 12: 593–594. 10.1167/12.1.25 22281692

[4] Kuzinas A. The stability of emotional associations of basic image attributes. Perception 2013; 42: 191.

[5] Truelove HB. Energy source perceptions and policy support: Image associations, emotional evaluations, and cognitive beliefs. Energy Policy 2012; 45: 478–489.

[6] Li HF, He HP, Chen JJ. A multi-layer affective model based on personality, mood and emotion. Journal of Computer-Aided Design & Computer Graphics 2011; 23: 725–729.

[7] Wang XH, Jia J, Tang J, Wu BY, Cai LH, Xie LX. Modeling emotion influence in image social networks. IEEE Transactions on Affective Computing 2015; 6: 286–297.

[8] Cho SB, Lee JY. A human-oriented image retrieval system using interactive genetic algorithm. IEEE Trans on Systems, Man and Cybernetics 2002; 32: 452–458.

[9] Liu Y, Chen X, Zhang CC, Sprague A. Semantic clustering for region-based image retrieval. J Vis Commun Image R 2008; 20: 157–166.

[10] Li PT, Shi YX, Dai HG. Classification of house-designing image based on color feature. Computer Engineering 2011; 37: 224–226, 229.

[11] Li ZH, Fan YY, Liu WH, Yu ZQ, Wang FQ. Emotional textile image classification based on cross-domain convolutional sparse autoencoders with feature selection. Journal of Electronic Imaging 2017; 26: 013022.

[12] Zhang H, Gönen M, Yang ZR, Erkki Oja. Understanding emotional impact of images using Bayesian multiple kernel learning. Neurocomputing 2015; 165: 3–13.

[13] Cao JF, Chen LC. Fuzzy Emotional Semantic Analysis and Automated Annotation of Scene Images. Computational intelligence and neuroscience 2015; 3: 971039. 10.1155/2015/971039 PMC4369949 25838818

[14] Xing BX, Zhang KJ, Sun SQ, Zhang LK, Gao ZG, Wang JX, et al Emotion-driven Chinese folk music-image retrieval based on DE-SVM. Neurocomputing 2015; 148: 619–627.

[15] Chang JK, Ryoo ST. Real-time emotion retrieval scheme in video with image sequence features. Journal of Real-Time Image Processing 2014; 9: 541–547.

[16] Yun T, Guan L. Human emotional state recognition using real 3D visual features from Gabor library. Pattern Recognition 2013; 46: 529–538.

[17] Mohamed H, Marchand-Maillet S. MRO-MPI: MapReduce overlapping using MPI and an optimized data exchange policy. Parallel Computing 2013; 39: 851–866.

[18] Chen C, Liu Z, Lin WH, Wang K. Distributed Modeling in a MapReduce Framework for Data-Driven Traffic Flow Forecasting. IEEE Transactions on Intelligent Transportation Systems 2013; 14: 22–33.

[19] Kim Y, Shim K, Kim MS, Lee JS. DBCURE-MR: An efficient density-based clustering algorithm for large data using MapReduce. Information Systems 2014; 42: 15–35.

[20] Qian J, Lv P, Yue XD, Liu CH, Jing ZJ. Hierarchical attribute reduction algorithms for big data using MapReduce. Knowledge-based Systems 2015; 73: 18–31.

[21] Almeer MH. Cloud Hadoop mapreduce for remote sensing image analysis. Journal of Emerging Trends in Computing and Information Sciences 2012; 3: 637–644.

[22] Wiley K, Connolly A, Krughoff S. Astronomical image processing with Hadoop. Proceedings of the 20th Conference on Astronomical Data Analysis Software and Systems 2011; 2011: 93–96.

[23] Ortony A, Clore GL, Collins A. The Cognitive Structure of Emotions. Cambridge, UK: Cambridge University Press, 1988.

[24] Wiggins JS. The five-factor model of personality: theoretical perspective. New York: Guilford Press, 1996.

[25] Mehrabian A. Analysis of the big-five personality factors in term of the PAD temperament mode. Australian journal of Psychology 1996; 48: 86–92.

[26] Lindner A, Süsstrunk S. Semantic-Improved Color Imaging Applications: It Is All About Context. IEEE Transactions on Multimedia 2015; 17: 700–710.

[27] Chernov V, Alander J, Bochko V. Integer-based accurate conversion between RGB and HSV color spaces. Computers & Electrical Engineering 2015; 46: 328–337.

[28] SUN Database, http://groups.csail.mit.edu/vision/SUN/, 2012.

[29] Yang Z, Yao YD, Li XC, Zheng D. A TDMA-based MAC protocol with cooperative diversity. IEEE Communications Letters 2010; 14: 542–544.

[30] Cover T, Hart P. Nearest neighbor pattern classification. IEEE Trans on Information Theory 1967; 13: 21–27.

[31] Hable R. Universal consistency of localized versions of regularized kernel methods. Journal of Machine Learning Research 2013; 14: 153–186.

[32] Triguero I, Peralta D, Bacardit J, Garcia S, Herrera F. MRPR: A MapReduce solution for prototype reduction in big data classification. Neurocomputing 2015; 150: 331–345.

[33] Guo MW, Zhao YZ, Xiang YP, Zhang CB, Chen ZH. Review of object detection methods based on SVM. Control and Decision 2014; 29: 193–200.

[34] Wang D, Sun JZ, Li FC, Song T. Protein structure prediction based on parallel multi-class SVM. Application Research of Computers 2011; 28: 465–468.

[35] Hubers AJ, Heideman DAM, Burgers SA, Herder PJ, Rhodius RJ, Smit HJ, et al DNA hypermethylation analysis in sputum for the diagnosis of lung cancer: training validation set approach. British Journal of Cancer 2015; 112: 1105–1113. doi: 10.1038/bjc.2014.636 25719833 10.1038/bjc.2014.636 PMC4366885

[36] Bantis LE, Nakas CT, Reiser B. Construction of confidence regions in the ROC space after the estimation of the optimal Youden index-based cut-off point. Biometrics 2014; 70: 212–223. doi: 10.1111/biom.12107 24261514 10.1111/biom.12107

[37] Han W, Zhang XQ, Chen Y. MapReduce based image classification approach. Journal of Computer Applications 2014; 34: 1600–1603.

